# Legal Notes for Institutional Workers

**Published:** 1913-07-26

**Authors:** 


### LEGAL NOTES FOR INSTITUTIONAL WORKERS.

**(The Editor will be pleased to answer Legal queries in this column.)**

### The Diagnosis of Death.

Some curious and interesting researches by Dr. M. H. Kahn, of New York, may possibly turn out to have supplied the world of science with a new and infallible test of death. It is common knowledge that by the use of the ophthalmoscope the arteries and veins of the retina can be quite easily seen, and the pulsations of the circulating blood watched in some circumstances. The lenses of the ophthalmoscope magnify the apparent size of these vessels, which are the only ones in which the blood can be viewed in a human being. Dr. Kahn has devoted attention to the condition of these vessels after death, of which there is but little information available in medical literature. He finds that in a short time after death transverse interruptions are visible in the previously continuous columns of blood; especially in the larger veins near the optic disc. At first, movement of the head of the corpse, or pressure on the eyeball, suffices to cause these segmentations to vary or to disappear temporarily; but by about three hours after death it is impossible any longer to cause such variations. Dr. Kahn's explanation of this phenomenon ascribes the appearances to clotting in the vessels and shrinking of the clots thus formed. Occasionally the blood clots *en masse*, and then the segmentations do not appear; and they are frequently absent when there has been optic neuritis during life. If further observations should show that this segmentation in the retinal vessels is never seen during any form of suspended animation, but only after actual death, the medico-legal importance of Dr. Kahn's work may easily be enormous. His paper appears in the *Medical Record* of May 3, 1913.

### Endurance after Fatal Injuries.

The question of how far a man can travel with his throat cut so severely as to cause his death is manifestly important from a medico-legal standpoint. Dr. J. A. Goldsmid reports in the *Australasian Medical Gazette* the following extraordinary case. A man of about forty years of age was found lying dead in a field. His throat was cut, but only a few drops of blood could be found near the body. On the top of a hill three hundred yards away was found a patch of ground soaked with blood, and near by a blood-stained razor and razor case. There was no sign of a struggle anywhere, either on the ground or on the body. The cut in the throat was comparatively small, but had pierced the thyro-hyoid membrane: no important vessel was severed, nor were the tissues blanched. Dr. Goldsmid, unfortunately, was ordered merely to inspect the body, and so was unable to make absolutely certain how death took place. He suggests that probably death was due to suffocation from blood trickling down into the lungs. This is possible, but surely hæmoptysis would be inevitable in such an event; and some blood stains would have been found along the line of the suicide's journey from the hill-top. Another possibility is that death was due to embolism from some vein in which a clot had formed.

